# Theoretical Investigation of the Enantioselective Complexations between *pf*DHFR and Cycloguanil Derivatives

**DOI:** 10.3390/scipharm85040037

**Published:** 2017-11-21

**Authors:** Suriyawut Kulatee, Pisanu Toochinda, Anotai Suksangpanomrung, Luckhana Lawtrakul

**Affiliations:** 1 School of Bio-Chemical Engineering and Technology, Sirindhorn International Institute of Technology, Thammasat University, Pathum Thani 12121, Thailand; suriyawut.kulatee@gmail.com (S.K.); pisanu@siit.tu.ac.th (P.T.); 2 Department of Mechanical Engineering, Academic Division, Chulachomklao Royal Military Academy, Nakhon Nayok 26001, Thailand; asuksang@hotmail.com

**Keywords:** antimalarial drugs, cycloguanil enantiomers, molecular docking, protein–ligand interaction

## Abstract

Point mutations in *Plasmodium falciparum* dihydrofolate reductase (*pf*DHFR), especially the double mutant variant (A16V + S108T), led to ineffective inhibiting by cycloguanil (Cyc). Cycloguanil derivatives showed good inhibiting properties against wild-type and mutant *pf*DHFR with an inhibition constant as low as the nanomolar level. However, there have been no reports on the stereochemistry of the compounds, and this is important because the pure enantiomeric form of a chiral drug can exert desirable, as well as non-desirable responses on the body or both. In this work, three-dimensional structures of Cyc derivatives in *R* and *S* configuration were constructed and optimized using Hartree-Fock/6-31G (d,p). Their structures were docked into the binding pocket of wild-type and double mutant (A16V + S108T) *pf*DHFR, complexed with nicotinamide adenine dinucleotide phosphate (NADPH). Results indicate that both wild-type and mutant *pf*DHFR are enantioselective towards enantiomeric Cyc derivatives (*R* and *S* configuration).

## 1. Introduction

**Figure 1:**
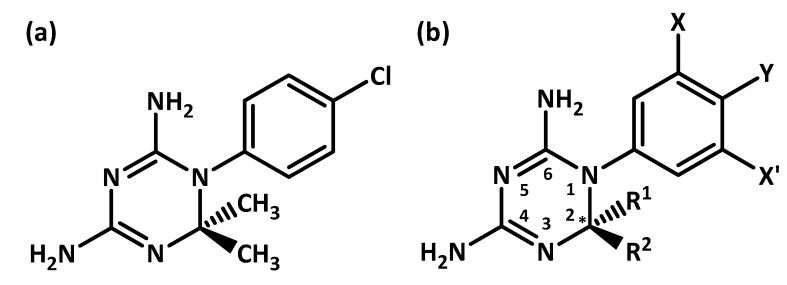
Chemical structure of (**a**) cycloguanil (Cyc) and (**b**) Cyc derivatives. Asterisk indicates chiral center. X and X’ are *m-*positions. Y is *p-*position.

*Plasmodium falciparum* dihydrofolate reductase (*pf*DHFR) is a key enzyme responsible for the *Plasmodium* parasite’s reproductive cycle. Point mutations at amino acid residues 16, 51, 59, 108, and 164 prevent the effective binding of antifolate treatments [1,2]. Among them, double mutations at residue 16 (alanine mutates to valine) and at residue 108 (serine mutates to threonine) confer cycloguanil (Cyc) resistance in the double mutant variant *pf*DHFR (A16V + S108T) [1,2]. To tackle this problem, Cyc derivatives are designed and experimentally tested against both wild-type and mutant *pf*DHFR (A16V + S108T). The new design of Cyc derivatives contain modifications at the C-2 and N-1 position (refer to Figure 1) [3,4]. The effects of changing the substituent at C-2 and the shifting of *p-*chlorophenyl to the *m-*position at N-1, results in the lowering of the inhibition constant (*K_i_*) between the Cyc derivative and mutant *pf*DHFR at the nanomolar level [5]. However, there have been no reports on the stereochemistry of the compounds, and this is important because the pure enantiomeric form of a chiral drug can exert desirable or non-desirable responses on the body or both [6,7,8]. Treating patients with the racemic drug (equimolar mixture of pure enantiomers: *R*- and *S*-enantiomers) can mitigate the disease but might cause minor to severe side effects at the same time [6]. For some chiral drugs, only one enantiomer is effective. This would, in theory, only require half of the effective dose of a 50:50 racemic mixture [6]. For example, the treatment of tuberculosis with racemic ethambutol can fight tuberculosis infections (*S,S*-(+)-ethambutol), but also causes optic nerve inflammation (*R,R*-(−)-ethambutol). The pure enantiomeric form of a chiral drug that exerts desirable effects and non-desirable effects is called a eutomer and a distomer, respectively. Taking a mixture of a eutomer and a distomer (in the form of racemates) may lead to different biological responses like: (i) distomer is inactive when compared to eutomer; (ii) distomer has the same biological activity as eutomer; (iii) distomer is less potent than eutomer; (iv) distomer acts as an antagonist to eutomer; (v) distomer exerts an adverse effect on eutomer; and (vi) distomer exerts different therapeutic effects than eutomer [6]. 

The general structures of Cyc and its derivatives are shown in Figure 1a,b, respectively. Cyc consists of a 1,3,5-dihydrotriazine ring with a 2,2-dimethyl substitution at the C-2 position and *p-*chlorophenyl substitution at the N-1 position. At the N-1 position of Cyc derivatives, the chlorophenyl substituents are substituted with either *p-* or *m-*chlorophenyl to avoid steric hindrance with Thr108 of mutant *pf*DHFR. At the C-2 position of Cyc derivatives, the dimethyl groups are substituted with either alkyl chains or phenol chains (R^1^ and R^2^) to avoid steric hindrance with Val16 of mutant *pf*DHFR. The substitution of flexible substituents at C-2 gives rise to one carbon chiral center. As a result, Cyc derivatives can exist as *R-* or *S-*enantiomers. Wild-type and mutant *pf*DHFR are also enantiomers because of the presence of chiral centers in their amino acid residues. Until date, there is no report on the stereochemistry of the Cyc derivatives and the authors are interested in studying the enantioselectivity of both the wild-type and double mutant *pf*DHFR with pure enantiomeric Cyc derivatives (the *R-* and *S-*enantiomers). 

## 2. Materials and Methods

The three-dimensional structures of Cyc and its derivatives are constructed using GaussView 5 [9]. The geometry of Cyc derivatives are optimized by Gaussian 09 using the basis set Hartree-Fock/6-31G (d,p), gaseous phase [10]. The x-ray crystal structures of wild-type *pf*DHFR (PDB ID: 3UM8 [11]) and double mutant (A16V + S108T) *pf*DHFR (PDB ID: 3UM6 [12]) are downloaded from the RCSB Protein Data Bank. Hydrogen atoms are added and water molecules are removed from the structures of the wild-type and double mutant *pf*DHFR via Discovery Studio Visualizer 4.0 [13]. Optimized ligands are then docked into the binding pockets of both wild-type and mutant *pf*DHFR via AutoDock 4.2 [14]. Ligands are kept flexible, while the enzyme macromolecules are kept rigid. Gasteiger charges are assigned to the system before performing molecular docking simulation. A grid size of 60 × 60 × 60 with 0.375 Å spacing is used. The dimensions and coordinates of grid boxes are kept constant throughout the docking process. One hundred docking calculations are performed on each ligand-enzyme complex using the Lamarckian genetic algorithm with remaining parameters run at default settings [15]. The results obtained are classified into different clusters with different binding energies. The cluster with the highest frequency and that also satisfies the essential binding characteristics is selected for further analysis. 

## 3. Results and Discussion

The binding energies (BE) of Cyc derivatives with the wild-type *pf*DHFR (3UM8) and mutant (A16V + S108T) *pf*DHFR (3UM6), and experimental BE are summarized in Table 1. The experimental BE are calculated from inhibition constant value, *K_i_* (taken from ref [5]). Their values are calculated using the formula: BE = −RTln(*K*) = RTln(*K_i_*), T = 298.15 K, and R = 8.314 JK^−1^ mol^−1^, where *K* and *K_i_* are the equilibrium constant and the inhibition constant (the reciprocal of *K*), respectively. The details of substituents (R^1^ and R^2^), chlorophenyl substitution at *m-*position (X) or *p*-position (Y), and experimental BE of Cyc derivatives are taken from [5].

**Figure 2:**
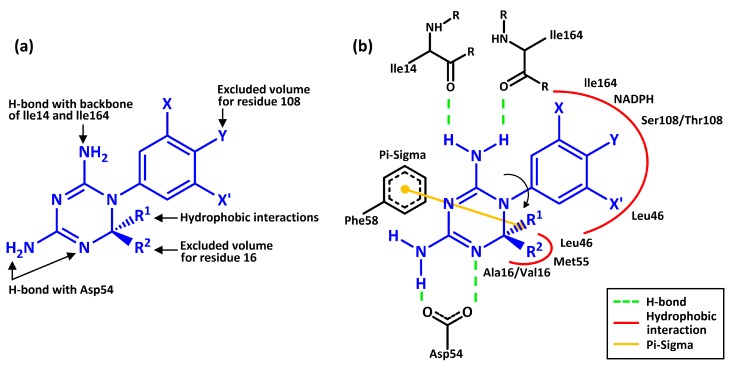
Simplified view of (**a**) essential binding characteristics of a good inhibitor of *pf*DHFR; (**b**) binding interactions of Cyc derivatives inside the wild-type and mutant *pf*DHFR binding pockets. Blue indicates the general structure of Cyc derivatives.

**Figure 3:**
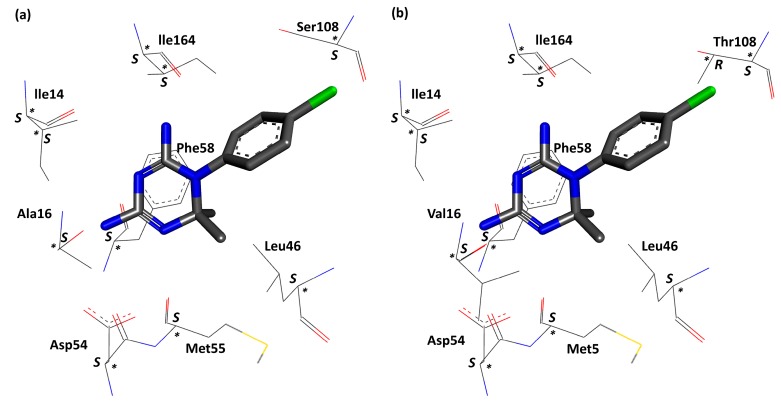
Chirality in the binding pocket of (**a**) wild-type *pf*DHFR; (**b**) double mutant (A16V + S108T) *pf*DHFR. Dark grey, blue, red, green, and yellow represents carbon, nitrogen, oxygen, chlorine, and sulfur atoms, respectively. Chiral center is indicated by asterisk.

The molecular docking results in Table 1 are selected according to the guideline of essential binding characteristics of a good *pf*DHFR inhibitor (Figure 2a,b) [16]. The chirality of amino acid residues in both the wild-type and mutant *pf*DHFR binding pocket is shown in Figure 3. Cycloguanil derivatives with the best-fit configuration have similar binding interactions to that in Figure 2b. For some Cyc derivatives, poor conformation exists inside the *pf*DHFR binding pocket. They occur because of the steric hindrance between Cyc derivatives and the side chain of amino acids in *pf*DHFR binding pocket. Cycloguanil derivatives with poor conformations do not have the best-fit configuration in their hundred docking frequencies. Cycloguanil derivatives with poor conformations have binding interactions different from the reference structure but they are able to meet some of the essential binding characteristics. For analysis purpose, the cluster with the highest frequency is selected. The best-fit configuration is selected from the cluster with highest frequency as well. For Cyc derivatives where both *R* and *S* configurations are the best-fit configuration, their binding interactions are compared based on the priority assigned in this order: (i) first priority is assigned to the strength of hydrogen bonding with Asp54 side chains because Asp54 is responsible for *pf*DHFR catalytic activity [16]; (ii) if Cyc derivatives have the same hydrogen bonding strength for Asp54 or they do not interact with Asp54, then the strength of hydrogen bonds of Cyc derivatives with Ile14 and Ile164 side chains are compared; (iii) hydrophobic interactions with residue 16 and 108 increase the overall binding affinity of that Cyc derivative; (iv) hydrophobic interaction of *m-*Cl with Leu46 decreases the BE of that configuration (*p-*Cl does not interact with Leu46).

*R-* and *S*-Cyc derivatives differ by the substituent priority at C-2 position. Their 1,3,5-dihydrotriazine rings and chlorophenyl rings are preserved. The most important part of Cyc derivatives is the 1,3,5-dihydrotriazine ring because hydrogen bondings are formed here (refer to Figure 2). Hydrophobic interactions are formed around the chlorophenyl ring at N-1 and the flexible substituents’ side chains at C-2 position. As a result, *R-* and *S*-Cyc derivatives can fit inside the binding pocket of both the wild-type and mutant *pf*DHFR. 

For molecular docking of wild-type, the charges of the molecule were derived from quantum mechanics (QM) Hartree-Fock/6-31G (d,p) and Gasteiger calculations. The results from both calculations are highly correlated with each other with R^2^ = 0.80 (Supplementary Materials).

**Figure 4:**
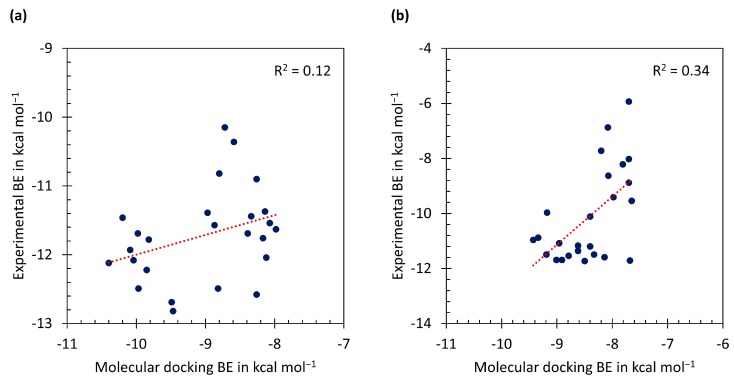
Plot of experimental binding energies (BE) versus molecular docking BE (Gasteiger charges) of (**a**) wild-type *pf*DHFR and (**b**) mutant *pf*DHFR.

Figure 4 presents the relationships of binding energy between the molecular docking calculations and the experimental data. The linear correlation coefficient (R^2^) between molecular docking and the experimental data are rather low for both the wild-type (Figure 4a) and mutant *pf*DHFR (Figure 4b). This is due to the position of all amino acid residues in the binding pockets are fixed during the molecular docking calculations. However, if we consider biological activity values of compounds within the same methodology, we are able to classify the potent and non-potent Cyc derivatives the same as the experimental data. In order to obtain the docking-based binding energies that are sufficiently accurate to discriminate the preferred ligand stereochemistry, more accurate methods for binding energy prediction as well as incorporating protein flexibility may be required to improve the quality of the predicted binding energies. These could be done by molecular dynamics simulations (MD) in the real aqueous environment. 

In experimental data, wild-type *pf*DHFR have higher binding affinity for Cyc derivatives with *p*-chlorophenyl than the *m*-chlorophenyl (except Cyc41–46). While few Cyc derivatives of the molecular docking results (3UM8) with the *p-*chlorophenyl (Cyc28, 30, and 32) have better binding activity towards wild-type *pf*DHFR. Wild-type *pf*DHFR can bind to Cyc derivatives with both the *p-* and *m*-chlorophenyl because residue size of Ser108 is smaller than Thr108 of the mutant *pf*DHFR (refer to Figure 3). As a result, both *p-* and *m*-Cl have more space to occupy and do not experience steric hindrance with Ser108.

**Figure 5:**
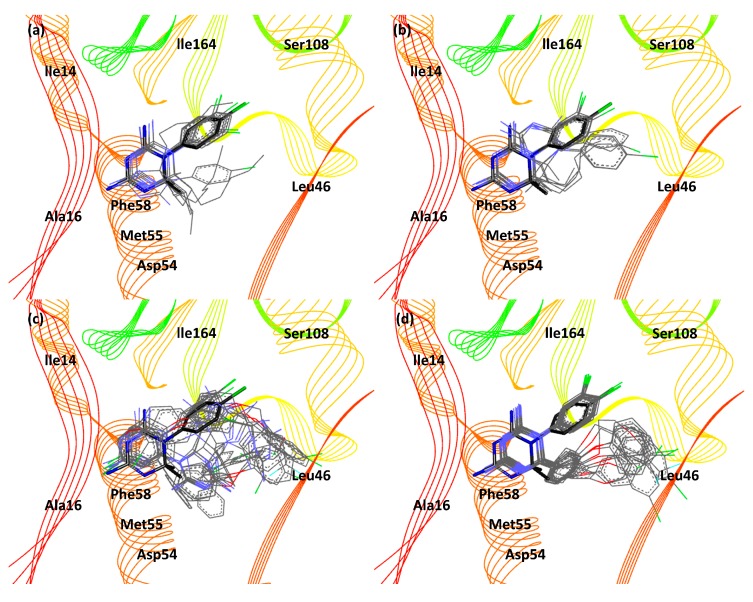
Superposition image of Cyc derivatives (*p-* and *m-*chlorophenyl substituent) with the reference structure in the wild-type *pf*DHFR binding pocket. Cyc24–31 and Cyc42 (R^2^ is alkyl chain) in (**a**) *R* configuration and (**b**) *S* configuration. Cyc32–41 and Cyc43–46 (R^2^ is phenol chain) in (**c**) *R* configuration and (**d**) *S* configuration. Cycloguanil derivatives and the reference structure are shown as line model and stick model, respectively. Black, blue, and green indicates carbon, nitrogen, and chlorine atom, respectively.

The results in 3UM8 indicate that Cyc24–27, Cyc30–33, and Cyc42 have better binding activity towards *pf*DHFR when they are in the *R* configuration. Cyc28–29, Cyc34–41, and Cyc43–46 bind better to *pf*DHFR when they are in the *S* configuration. Structural analysis of Cyc derivatives reveals that Cyc derivatives with alkyl chains (except Cyc28 and 29) are preferred for the *R*-enantiomer and Cyc derivatives with phenol chains (except Cyc32 and 33) are preferred for the *S*-enantiomer. *R-*Cyc derivatives with alkyl chains have better binding activity than *S-*Cyc derivatives because they can avoid steric hindrance with the Phe58 side chains. *S*-Cyc derivatives with phenol chains have better binding activity than *R*-Cyc derivatives because they can avoid steric hindrance with the Leu46 and Met55 side chains. Cyc28, 29, 32, and 33 are exceptions because the size of their substituents is the transition between non-bulky alkyl chains and bulky phenol chains. The superposition image of Cyc derivatives (line model) with the reference structure (stick model) in the wild-type *pf*DHFR binding pocket is shown in Figure 5. Good superposition is observed in Figure 5a,d. Cyc31 and 33 experience steric hindrance with the Ile164 side chains, resulting in the chlorophenyl ring rotation from position X to X’, as seen in Figure 5a. 

**Figure 6:**
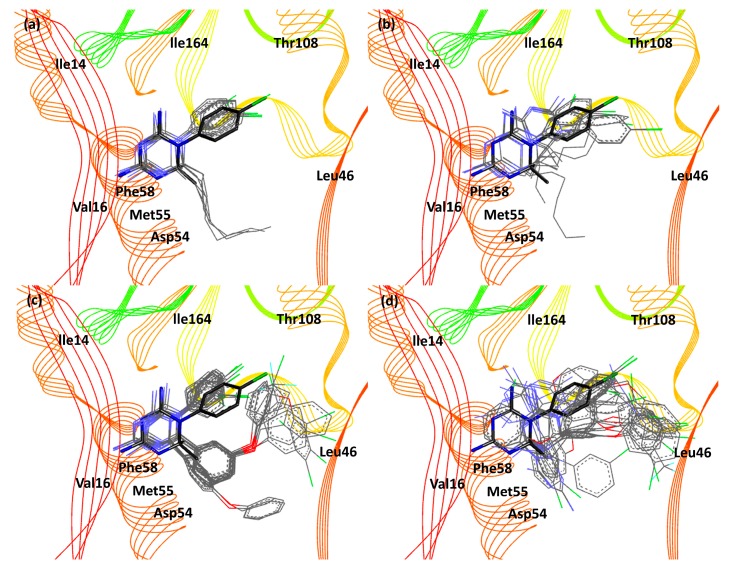
Superposition image of Cyc derivatives (*p-* and *m-*chlorophenyl substituent) with the reference structure in the mutant *pf*DHFR binding pocket. Cyc24–31 and Cyc42 (R^2^ is alkyl chain) in (**a**) *R* configuration and (**b**) *S* configuration. Cyc32–41 and Cyc43–46 (R^2^ is phenol chain) in (**c**) *R* configuration and (**d**) *S* configuration. Cycloguanil derivatives and the reference structure are shown as line model and stick model, respectively. Black, blue, and green indicates carbon, nitrogen, and chlorine atom, respectively.

Mutant *pf*DHFR BE values have similar trend to the experimental data as well. Cycloguanil derivatives, irrespective of the substituent type, are preferred for the *R-*enantiomer. Mutant *pf*DHFR is made up of chiral centers. The chirality within mutant *pf*DHFR are similar to that of wild-type, except for Thr108 that contains two chiral centers (*R* and *S* configuration). The highest available enantiomers are in *S* configuration (refer to Figure 3). The increase in the bulkiness of Val16 and Thr108 results in the reduction of binding pocket volume around them. Mutation at residue 108 results in Cyc derivatives with *p-*Cl (except Cyc28, 34, 36, 38, and 40) to experience steric hindrance with Thr108 side chains. Val16 is situated in front Phe58 (refer to Figure 3). Because Val16 is bulkier than Ala16, the pocket volume Val16 and Phe58 is reduced, resulting in the *R-*Cyc derivatives to have better binding activity than the *S-*Cyc derivatives. Chlorophenyl ring rotation (Cyc25, 27, 29, 31, 33, 35, 42, and 43) is observed in mutant *pf*DHFR as well (refer to Figure 6a,c). 

## 4. Conclusions

Theoretical investigation of the enantioselective complexations between wild-type and mutant *pf*DHFR and Cyc derivatives shows that both *pf*DHFR can bind to Cyc derivatives in *R* and *S* configuration. In wild-type *pf*DHFR, *R-*Cyc derivatives with alkyl chains (except Cyc28 and 29) are preferred over the *S-*Cyc derivatives because they do not experience steric hindrance with Phe58 side chains. *S-*Cyc derivatives with phenol chains (except Cyc32 and 33) are preferred over *R-*Cyc derivatives because they do not experience steric hindrance with the Leu46 and Met55 side chains. Cycloguanil derivatives with *p-* and *m-*chlorophenyl rings can form hydrophobic interaction with the wild-type *pf*DHFR due to larger binding pocket volume. In mutant *pf*DHFR, *R-*Cyc derivatives, irrespective of the substituent type, are preferred over the *S*-Cyc derivatives because they do not experience steric hindrance with Phe58 side chains. Val16 in the mutant *pf*DHFR is bulkier than Ala16 in the wild-type. As a result, the pocket volume around Val16 and Phe58 is reduced, resulting in the flexible side chains of *S-*Cyc derivatives to experience steric hindrance with the Phe58 side chains. Cycloguanil derivatives with *m*-Cl are preferred over *p-*Cl because Thr108 in mutant *pf*DHFR is bulkier than Ser108 of the wild-type, resulting in Cyc derivatives with *p*-Cl to experience steric hindrance with Thr108 side chains. The effect of chlorophenyl ring rotation to avoid steric hindrance with Ile164 side chain, is observed in both the wild-type and mutant *pf*DHFR. In addition, the substitution of *p-* and *m-*Cl in Cyc derivatives do not affect the enantiomeric form of Cyc derivatives. 

## Figures and Tables

**Table 1:** Binding energy of Cyc derivatives (kcal mol^–1^) for binding with the wild-type *Plasmodium falciparum* dihydrofolate reductase (*pf*DHFR) (3UM8) and mutant (A16V + S108T) *pf*DHFR (3UM6) obtained from molecular docking calculations and experimental data.

					3UM8			3UM6		
Comp.	X	Y	R^1^	R^2^	*R*	*S*	Exp.	*R*	*S*	Exp.
Cyc	H	Cl	Me	Me	−8.12 −7.98		−12.04	−7.70 −7.70		−8.02
**23**	Cl	H	Me	Me			−11.63			−8.88
**24**	H	Cl	Me	*n*Pr	**−8.07**	−6.85 ^b^	−11.54	**−8.08**	−7.09 ^b^	−6.87
**25**	Cl	H	Me	*i*Pr	**−8.59**	−7.30 ^b^	−10.36	**−8.20 ^a^**	−7.41 ^b^	−7.72
**26**	H	Cl	Me	*i*Pr	**−8.72**	−7.12 ^b^	−10.15	**−7.70**	−7.37 ^b^	−5.93
**27**	Cl	H	Me	*n*Pr	**−8.14**	−8.01	−11.37	**−8.07**	−7.57 ^a^	−8.63
**28**	H	Cl	Me	*n*Hex	−7.75 ^b^	**−8.26**	−12.58	**−7.81**	−7.79	−8.21
**29**	Cl	H	Me	*n*Hex	−7.85	**−8.17**	−11.76	**−7.65 ^a^**	−7.62 ^a^	−9.54
**30**	H	Cl	H	Me	**−8.34**	−7.76	−11.44	**−7.98**	−7.53	−9.41
**31**	Cl	H	H	Me	**−8.26 ^a^**	−7.83	−10.90	**−8.40 ^a^**	−7.48	−10.11
**32**	H	Cl	H	C_6_H_5_	**−8.97**	−8.39	−11.39	**−9.18**	−7.21 ^b^	−9.97
**33**	Cl	H	H	C_6_H_5_	**−8.80 ^a^**	−8.67	−10.82	**−9.34 ^a^**	−7.19	−10.88
**34**	H	Cl	H	4-C_6_H_5_OC_6_H_5_	−8.57 ^b^	**−9.47**	−12.82	**−9.19**	−9.04 ^b^	−11.49
**35**	Cl	H	H	4-C_6_H_5_OC_6_H_5_	−8.47 ^b^	**−9.97**	−12.49	**−9.01 ^a^**	−7.22 ^b^	−11.69
**36**	H	Cl	H	3-C_6_H_5_OC_6_H_5_	−8.60	**−9.49**	−12.69	**−8.91**	−6.81	−11.69
**37**	Cl	H	H	3-C_6_H_5_OC_6_H_5_	−8.73 ^b^	**−9.85**	−12.22	−8.50	**−8.55 ^b^**	−11.73
**38**	H	Cl	H	3-C_6_H_5_CH_2_OC_6_H_4_	−8.12	**−8.82**	−12.49	**−8.40**	−6.74	−11.20
**39**	Cl	H	H	3-C_6_H_5_CH_2_OC_6_H_4_	−9.31 ^b^	**−9.82**	−11.78	**−8.14**	−7.01	−11.59
**40**	H	Cl	H	3-(4-ClC_6_H_4_O)C_6_H_4_	−8.82	**−10.04**	−12.08	**−8.96**	−7.27	−11.08
**41**	Cl	H	H	3-(4-ClC_6_H_4_O)C_6_H_4_	−9.25 ^b^	**−10.40**	−12.12	**−8.79**	−7.39	−11.54
**42**	Cl	H	H	*n*C_7_H_15_	**−8.39 ^a^**	−8.03	−11.69	**−8.33 ^a^**	−7.59	−11.49
**43**	Cl	H	H	4-PrOC_6_H_4_	−7.43 ^b^	**−8.87**	−11.57	**−9.43 ^a^**	−8.05 ^b^	−10.96
**44**	Cl	H	H	3-(3,5-Cl_2_C_6_H_3_O)C_6_H_4_	−8.90 ^b^	**−10.09**	−11.93	**−8.62**	−6.93 ^b^	−11.36
**45**	Cl	H	H	3-[2,4,5-Cl_3_C_6_H_2_O(CH_2_)_3_O]C_6_H_4_	−9.18 ^b^	**−10.20**	−11.46	**−7.68**	−3.49 ^b^	−11.71
**46**	Cl	H	H	3-(3-CF_3_C_6_H_4_O)C_6_H_4_	−8.20 ^b^	**−9.98**	−11.69	**−8.62**	−6.74	−11.17

^a^ *m-*Cl at X flips to X’ position; ^b^ Poor conformation; X: *m-*position; Y: *p*-position; R^1^, R^2^: substituents; *R:* Cyc derivatives in *R* configuration; *S*: Cyc derivatives in *S* configuration; Exp.: Experimental data; Comp.: Compound; Bold: Enantiomer configuration with the lowest BE.

## Supplementary Materials

The following are available online at http://www.mdpi.com/2218-0532/85/4/37/s1, Table S1: Binding energy (BE) of Cyc derivatives (kcal mol^−1^) for binding with the wild-type *pf*DHFR (PDB ID: 3UM8), using derived quantum mechanics (QM) Hartree-Fock/6-31G (d,p) charges and Gasteiger charges obtained from molecular docking calculations and experimental data; Figure S1: Plot of molecular docking binding energies (BE) of (**a**) *R-*Cyc derivatives using QM Hartree-Fock/6-31G (d,p) charges versus *R*-Cyc derivatives using Gasteiger charges; (**b**) *S-*Cyc derivatives using QM Hartree-Fock/6-31G (d,p) charges versus *S*-Cyc derivatives using Gasteiger charges.
