# Supplementary material for: Theoretical Investigation of the Enantioselective Complexations between pfDHFR and Cycloguanil Derivatives
Source: Sci Pharm. 2017 Nov 21;85(4):37. doi: 10.3390/scipharm85040037 (PMC5748534; doi:10.3390/scipharm85040037)

Supplementary Materials

Theoretical Investigation of the Enantioselective Complexations between *pf*DHFR and Cycloguanil Derivatives

Table S1. Binding energy (BE) of Cyc derivatives (kcal mol^{-1}) for binding with the wild-type *pf*DHFR (PDB ID: 3UM8), using derived quantum mechanics (QM) Hartree-Fock/6-31G (d,p) charges and Gasteiger charges obtained from molecular docking calculations and experimental data.

					**QM**		**Gasteiger**		**Exp.**
**Com**	**X**	**Y**	**R^1**	**R^2**	***R***	***S***	***R***	***S***	
**23**	Cl	H	Me	Me	-5.09		-7.98		-11.63
**24**	H	Cl	Me	*n*Pr	**-4.92**	-4.26	**-8.07**	-6.85	-11.54
**25**	Cl	H	Me	*i*Pr	**-5.56**	-4.76	**-8.59**	-7.30	-10.36
**26**	H	Cl	Me	*i*Pr	**-5.59**	-4.71	**-8.72**	-7.12	-10.15
**27**	Cl	H	Me	*n*Pr	**-5.11**	-5.03	**-8.14**	-8.01	-11.37
**28**	H	Cl	Me	*n*Hex	-4.97	**-5.17**	-7.75	**-8.26**	-12.58
**29**	Cl	H	Me	*n*Hex	-5.22	**-5.31**	-7.85	**-8.17**	-11.76
**30**	H	Cl	H	Me	**-5.29**	-4.77	**-8.34**	-7.76	-11.44
**31**	Cl	H	H	Me	**-5.22**	-4.83	**-8.26**	-7.83	-10.90
**32**	H	Cl	H	C_6H_5	**-6.29**	-6.16	**-8.97**	-8.39	-11.39
**33**	Cl	H	H	C_6H_5	**-6.57**	-6.43	**-8.80**	-8.67	-10.82
**34**	H	Cl	H	4-$C_6H_5OC_6H_5$	-6.18	**-6.38**	-8.57	**-9.47**	-12.82
**35**	Cl	H	H	4-$C_6H_5OC_6H_5$	-6.04	**-6.73**	-8.47	**-9.97**	-12.49
**36**	H	Cl	H	3-$C_6H_5OC_6H_5$	-6.26	**-6.46**	-8.60	**-9.49**	-12.69
**37**	Cl	H	H	3-$C_6H_5OC_6H_5$	-6.18	**-6.67**	-8.73	**-9.85**	-12.22
**38**	H	Cl	H	3-$C_6H_5CH_2OC_6H_4$	-6.67	**-6.74**	-8.12	**-8.82**	-12.49
**39**	Cl	H	H	3-$C_6H_5CH_2OC_6H_4$	-6.22	**-6.67**	-9.31	**-9.82**	-11.78
**40**	H	Cl	H	3-(4-ClC_6H_4O)C_6H_4	-7.06	**-7.19**	-8.82	**-10.04**	-12.08
**41**	Cl	H	H	3-(4-ClC_6H_4O)C_6H_4	-6.75	**-7.58**	-9.25	**-10.40**	-12.12
**42**	Cl	H	H	*n*C_7H_{15}	**-6.02**	-6.01	**-8.39**	-8.03	-11.69
**43**	Cl	H	H	4-$PrOC_6H_4$	-5.09	**-5.93**	-7.43	**-8.87**	-11.57
**44**	Cl	H	H	3-(3,5-$Cl_2C_6H_3O$)C_6H_4	-6.84	**-7.63**	-8.90	**-10.09**	-11.93
**45**	Cl	H	H	3-[2,4,5-$Cl_3C_6H_2O(CH_2)_3O$]C_6H_4	-6.34	**-7.19**	-9.18	**-10.20**	-11.46
**46**	Cl	H	H	3-(3-$CF_3C_6H_4O$)C_6H_4	-5.13	**-5.65**	-8.20	**-9.98**	-11.69

QM	=	Molecular docking using Hartree-Fock/6-31G (d,p) charges
Gasteiger	=	Molecular docking using Gasteiger charges
Exp.	=	Experimental data
R	=	Cyc derivatives in *R* configuration
S	=	Cyc derivatives in *S* configuration
Bold	=	Enantiomer configuration with the lowest BE

Figure S1. Plot of molecular docking binding energies (BE) of (**a**) *R*-Cyc derivatives using QM Hartree-Fock/6-31G (d,p) charges *versus* *R*-Cyc derivatives using Gasteiger charges; (**b**) *S*-Cyc derivatives using QM Hartree-Fock/6-31G (d,p) charges *versus* *S*-Cyc derivatives using Gasteiger charges.

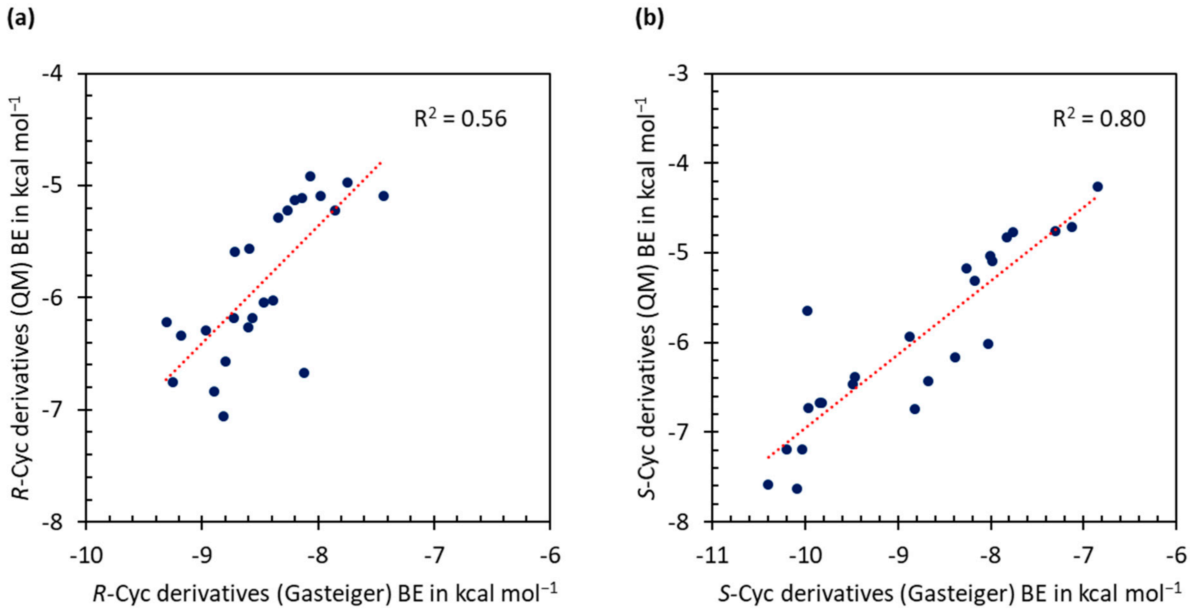

Supplement: Supplementary file 1 [file scipharm-85-00037-s001.pdf]
